# Commodity Wi-Fi-Based Wireless Sensing Advancements over the Past Five Years

**DOI:** 10.3390/s24227195

**Published:** 2024-11-10

**Authors:** Hai Zhu, Enlai Dong, Mengmeng Xu, Hongxiang Lv, Fei Wu

**Affiliations:** School of Electronic and Electrical Engineering, Shanghai University of Engineering Science, Shanghai 201620, China; m325123302@sues.edu.cn (E.D.); m320123403@sues.edu.cn (M.X.); m325122241@sues.edu.cn (H.L.); 02100007@sues.edu.cn (F.W.)

**Keywords:** Wi-Fi sensing, CSI, commodity-off-the-shelf, integrated sensing and communication

## Abstract

With the compelling popularity of integrated sensing and communication (ISAC), Wi-Fi sensing has drawn increasing attention in recent years. Starting from 2010, Wi-Fi channel state information (CSI)-based wireless sensing has enabled various exciting applications such as indoor localization, target imaging, activity recognition, and vital sign monitoring. In this paper, we retrospect the latest achievements of Wi-Fi sensing using commodity-off-the-shelf (COTS) devices from the past 5 years in detail. Specifically, this paper first presents the background of the CSI signal and related sensing models. Then, recent studies are categorized from two perspectives, i.e., according to their application scenario diversity and the corresponding sensing methodology difference, respectively. Next, this paper points out the challenges faced by Wi-Fi sensing, including domain dependency and sensing range limitation. Finally, three imperative research directions are highlighted, which are critical for realizing more ubiquitous and practical Wi-Fi sensing in real-life applications.

## 1. Introduction

The demand for ubiquitous internet connection has catalyzed the vast deployment of Wi-Fi infrastructures over the past decades, making Wi-Fi signal available almost everywhere. With the rapid progress of wireless communication and signal processing techniques, researchers have successfully reused Wi-Fi as a sensing platform beyond its traditional use as a pure communication medium, which further gives birth to the idea of integrated sensing and communication (ISAC) with Wi-Fi [1,2,3]. After years of persistent research, Wi-Fi sensing is drawing huge attention from both academia and industry [4]. Both communities recognize ISAC as a compelling technology capable of improving spectrum efficiency and reducing the hardware cost [5]. It is worth mentioning that, starting from 2020, the IEEE 802.11 working group established an IEEE 802.11bf standardization group for encompassing wireless sensing within the new version of 802.11 standard, turning Wi-Fi sensing into reality.

The basic rational behind Wi-Fi sensing is quite straightforward [6]. When wireless signal propagates from the transmitter to the receiver through multiple paths, a phenomenon called multi-path effect occurs, whereby the superimposed receiving signal intrinsically contains the signal component reflected or diffracted by the sensing target. Therefore, by analyzing the target “modulated” receiving signals, researchers can recover the rich information regarding the target, such as location and activity. Compared with classic sensor-based and vision-based sensing paradigms, Wi-Fi wireless sensing has the advantages of low-cost ubiquity, wide coverage, non-intrusiveness, and privacy-protection. Due to its appealing superiority, numerous Wi-Fi sensing applications have been developed, ranging from coarse-grained motion detection [7] and activity recognition [8] to fine-grained localization [9] and breath monitoring [10].

Inspired by existing survey papers [11,12,13,14,15], this paper investigates the thrilling achievements made within the last 5 years and presents an in-depth analysis of these sensing systems, aiming to facilitate further research in the Wi-Fi sensing field. This paper first divides existing works according to different application scenarios, including localization and tracking, activity recognition, vital sign monitoring, and target imaging. For each category, both application-specific problems and solutions are compared and summarized. Then, this paper further classifies recent studies based on the methodology adopted, whether it is model-based, handcrafted pattern extraction-based, or deep learning-based, pointing out the pros and cons of each method. Furthermore, this paper highlights the remaining challenges of current works such as generalization issues and large-scale perception. Future research directions and the need for further study are discussed in the end. The main contributions of this work are summarized as follows:

- To the best of our knowledge, this is the latest comprehensive survey in the Wi-Fi sensing field, covering the greatest and most recent progresses made over the past 5 years.
- We categorize existing studies from two distinct perspectives, i.e., application-based and methodology-based, and present an in-depth analysis of recent works.
- We highlight the key challenges encountered in existing studies and present a thorough discussion about three promising research directions for Wi-Fi sensing.

The rest of this paper is organized as follows. In Section 2, we briefly introduce the concept of channel state information (CSI) and explain several popular sensing models. In Section 3, we classify state-of-the-art works with regard to two criteria, i.e., application variety and methodology difference. Practical limitations and challenges are analyzed in Section 4. In Section 5, a detailed discussion about future trends in Wi-Fi sensing is provided. Finally, we conclude this article in Section 6.

## 2. Preliminary

Before analyzing Wi-Fi sensing, we briefly introduce the necessary background of channel state information (CSI) and several general signal sensing models.

### 2.1. Channel State Information

Serving as a key metric of a communication system, CSI depicts how a signal propagates through a wireless channel. Indeed, a wireless communication channel can be defined as follows:

*Y* = *H* × *X* + *N* (1)

where *X* and *Y* are the transmitted and received signal, respectively. *H* is the channel matrix representing CSI and *N* denotes the channel noise.

**Figure 1:**
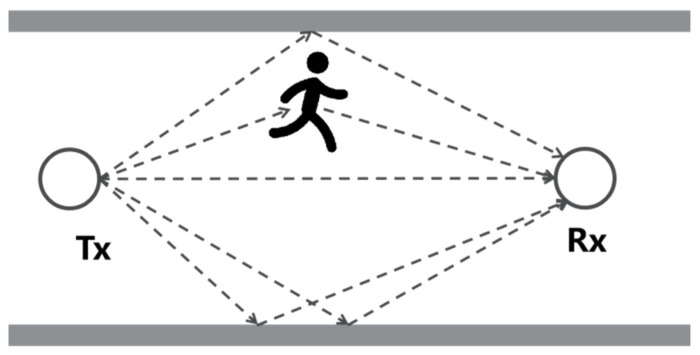
Typical indoor multi-path Wi-Fi propagation.

In a typical indoor environment, shown in Figure 1, a signal sent by the transmitter (Tx) travels through multiple paths before arriving at the receiver (Rx), which is also known as the multi-path effect. Therefore, assuming there are *L* different paths, the wireless channel *H* can be mathematically expressed as channel impulse response (CIR) [6], as follows:

(2) $$h\left(t\right)=\sum_{i=1}^{L}a_{i}e^{-j\theta_{i}}\delta \left(t-\tau_{i}\right)$$

where $a_{i}$, $\theta_{i}$, and $\tau_{i}$ are the complex amplitude attenuation, phase shift, and propagation time delay of the *i*-th path, respectively; $\delta \left(t\right)$ is the Dirac delta function. Each impulse in the summation of Equation (2) represents a delayed multi-path component, multiplied by its corresponding amplitude and phase variation.

As shown in Figure 1, when a person moves inside the signal zone, the human body will inevitably alter the specific propagation path, thus changing the CIR. Hence, the underlying principle of wireless sensing is analyzing human-induced channel variation. However, CIR cannot be precisely measured with commodity Wi-Fi devices, especially given the limited bandwidth of Wi-Fi. Fortunately, with the adoption of the orthogonal frequency division multiplex (OFDM) technique in present IEEE 802.11 standard, researchers resorted to studying channel frequency response (CFR), an equivalent channel representation of CIR in the frequency domain.

(3) $$CFR\left(f\right)=\left|CFR\left(f\right)\right|e^{j∠CFR\left(f\right)}$$

where $\left|CFR\left(f\right)\right|$ and $∠CFR\left(f\right)$ represent the amplitude–frequency and phase–frequency response of CFR, respectively. With proper driver modifications, researchers have been able to obtain an OFDM-based sampling version of CFR with a commercial-off-the-shelf (COTS) Wi-Fi network interface card (NIC) since 2010 [16,17], greatly prompting the development of Wi-Fi sensing [12]. To be specific, the extracted CFR depicts the amplitude and phase of different subcarriers, which can be expressed as follows:

(4) $$H\left(f_{i}\right)=\left|H\left(f_{i}\right)\right|e^{j∠H\left(f_{i}\right)}$$

where $H\left(f_{i}\right)$ is the CFR sampled at the *i*-th subcarrier with the central frequency of $f_{i}$. In fact, the CSI data $H=\left\{H\left(f_{i}\right)|i\in \left[1,\;N\right]\right\}$ used in most research papers refers directly to the definition given by Equation (4), i.e., a sampled version of CFR at the granularity of a subcarrier level.

Generally speaking, this sampled CFR lays the foundations for advanced Wi-Fi sensing, paving the way for the feasibility of various modern applications. CSI data contains rich information on signal propagation, and we will use CSI to simply signify the raw Wi-Fi data for brevity in the following part.

### 2.2. Signal Sensing Models

#### 2.2.1. Fresnel Zone-Based Reflection Model

**Figure 2:**
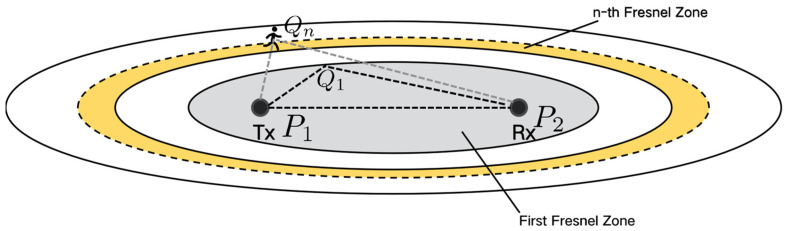
Geometry of Fresnel zone reflection sensing [18].

Taking one pair of the Tx-Rxlink as an example, Fresnel zones are concentric ellipses with two foci corresponding to the Tx and Rx, as *P*_1_ and *P*_2_, shown in Figure 2. For a given radio length λ, the *n*-th Fresnel zone boundary containing *n* ellipses can be defined as follows:

(5) $$\left|P_{i}Q_{n}\right|+\left|Q_{n}P_{2}\right|-\left|P_{1}P_{2}\right|=n\lambda /2$$

where $Q_{n}$ is a point on the *n*-th Fresnel zone boundary. The *n*-th Fresnel zone refers to the elliptic annulus between the (*n*−1)-th and *n*-th ellipse boundary, while the innermost ellipse is called the first Fresnel zone (FFZ). Equation (5) indicates that the path length of the signal reflected through the *n*-th Fresnel zone boundary is nλ/2 is longer than that of the Line-of-Sight (LOS) path, i.e., $\left|P_{1}P_{2}\right|$.

The Fresnel zone-based reflection model [18] characterizes how the amplitude and phase of CSI change when a target moves outside the FFZ. The key property of the reflection sensing model is when a target moves across a series of Fresnel zone boundaries, CSI amplitude and phase will show a continuous sinusoidal-like pattern, which can be utilized for sensing applications such as respiration and walking direction detection [19].

#### 2.2.2. Fresnel Zone-Based Diffraction Model

**Figure 3:**
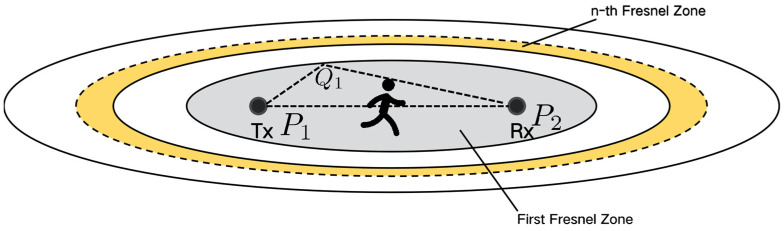
Geometry of Fresnel zone diffraction sensing [20].

According to the RF propagation theory, more than 70% of the signal energy is transferred via the FFZ. Therefore, when a target moves inside the FFZ, signal diffraction becomes more important and dominates the received signal variation. As shown in Figure 3, the Fresnel zone-based diffraction model [20] depicts how the amplitude and phase of CSI change when a target moves inside the FFZ. The key property is when sensing activity inside the FFZ, the CSI amplitude variation will show different shapes, be it either monotonically decreased or non-monotonous, “W”, depending on the target size. Apart from respiration monitoring, the diffraction sensing model has also been proved effective for recognizing exercise and daily exercise [8].

#### 2.2.3. Scattering Sensing Model

**Figure 4:**
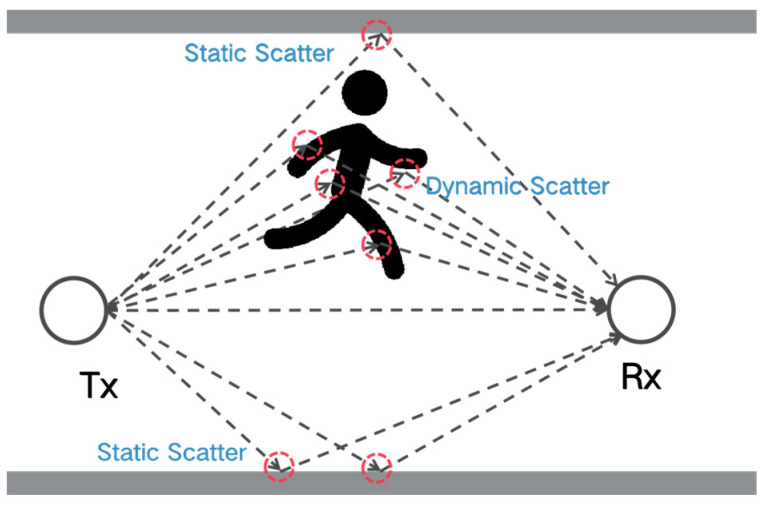
Signal scattering sensing model.

One main limitation of the previous models is that the simple reflection or diffraction assumption may not hold true when considering complex target motions, in cases where signals are scattered from multiple human body parts. Different from the Fresnel zone-based model, the scattering sensing model treats all objects as scatters, taking into account all multi-paths together. As marked as red circles in Figure 4, intuitively, the scattering model considers each scatter point as a virtual Tx, e.g., the static walls, and the arm and leg of the moving human. Given numerous multi-paths are considered, the scattering model is in fact a statistical model generally applicable to complex indoor scenarios. The scattering sensing model has been adopted in various speed-oriented tasks [21,22], achieving robust performance even with non-line-of-sight (NLOS) occlusion.

## 3. Wi-Fi Sensing

Serving as a key property in future wireless systems, Wi-Fi sensing has enabled various important applications. In this section, we categorize recent works based on two aspects, i.e., whether they are application-oriented or methodology-oriented. Since there are quite a few references in this section, for the reader’s convenience, we provide an index of all mentioned references in Table A1 of Appendix A.

### 3.1. Wi-Fi Sensing Applications

In this section, we divide the related works into seven categories, i.e., presence detection, gait recognition, gesture recognition, activity recognition, localization and tracking, vital sign monitoring, and pose construction and imaging, as shown in Table 1, Table 2, Table 3, Table 4, Table 5, Table 6 and Table 7. In each table, “Application” implies detailed application demand, “User number” signifies the number of sensing targets supported by the study, “Device type” indicates the specific sensing equipment used, and “NLOS” shows whether the sensing system can work in a non-line-of-sight scenario or not.

**Table 1:** Presence detection.

Year	Reference	Application	Performance	User Number	Device Type	NLOS
2022	WiCPD [23]	In-car child presence detection	96.56–100% real-time detection rate	1	NXP Wi-Fi chipset	Y
2023	Hu et al. [24]	Proximity detection	95% and 99% true positive rate for distance-based and room-based detection	1	NXP Wi-Fi chipset	Y
2024	Zhu et al. [25]	Human and non-human differentiation	95.57% average accuracy	1 human or pet	COTS device	Y
2024	WI-MOID [26]	Edge device-based human and non-human differentiation	97.34% accuracy and 1.75% false alarm rate	1 human or non-human subject	Wi-Fi edge device	Y

**Presence detection.** Presence detection determines whether a target exists or not within the sensing area and serves as the prerequisite for further sensing tasks. Target presence detection could enable many modern applications, such as security systems and smart homes. Although usually included as a detector module in most studies, there have been some new applications based on presence detection. As shown in Table 1, WiCPD [23] studied child presence detection in a smart car scenario, preventing potential harm to children if left alone in a vehicle. Hu et al. [24] considered target location relative to the sensing device, supporting more intelligent control systems using this area-aware context. In addition, Zhu et al. [25] and WI-MOID [26] further differentiated human from non-human targets to mitigate influence from unwanted objects, avoiding unnecessary false alarms.

**Table 2:** Gait recognition.

Year	Reference	Application	Performance	User Number	Device Type	NLOS
2021	GaitSense [27]	Gait-based human identification	93.2% for 5 users and 76.2% for 11 users	11	Intel 5300	N
2021	GaitWay [28]	Gait speed estimation	0.12 m median error	1	Intel 5300	Y
2022	CAUTION [29]	Gait-based human authentication	93.06 average accuracy	15	TP-Link N750 router	N
2022	Wi-PIGR [30]	Gait recognition	93.5% for single user and 77.15% for 50 users	1–50	Intel 5300	N
2023	Auto-Fi [31]	Gesture and gait recognition	86.83% for gesture; 79.61% for gait	1	Atheros chipset	N
2023	GaitFi [32]	Gait recognition	94.2% accuracy	12	TP-Link N750 router	N
2024	Wi-Diag [33]	Multi-subject abnormal gait diagnosis	87.77% average accuracy	4	Intel 5300	N

**Gait recognition.** Gait, a unique biomarker, refers to the distinctive walking character of different people and has been used for human identification and authentication applications. Early gait sensing works usually required users to walk on fixed trajectories within restricted areas, while recent studies, e.g., GaitSense [27], GaitWay [28], and Wi-PIGR [30], aimed for path independent gait recognition where users can walk along arbitrary paths even in a through-the-wall scenario. In addition, CAUTION [29], Auto-Fi [31], and GaitFi [32] tried to realize robust gait recognition with limited training data, while Wi-Diag [33] further studied more challenging multi-human recognition problems. As depicted in Table 2, all these works greatly contribute to more ubiquitous gait-based sensing applications.

**Table 3:** Gesture recognition.

Year	Reference	Application	Performance	User Number	Device Type	NLOS
2021	Kang et al. [34]	Gesture recognition	3–12.7% improvement	1	Widar Dataset	N
2021	WiGesture [35]	Gesture recognition	92.8–94.5% accuracy	1	Intel 5300	N
2022	HandGest [36]	Handwriting recognition	95% accuracy	1	Intel 5300	N
2022	DPSense-WiGesture [37]	Gesture recognition	94% average accuracy	1	Intel 5300	N
2022	Niu et al. [38]	Gesture recognition	96% accuracy	1	Intel 5300	Y
2022	Widar 3.0 [39]	Cross-domain gesture recognition	92.7% in-domain and 82.6–92.4% cross-domain accuracy	1	Intel 5300	N
2022	WiFine [40]	Gesture recognition	96.03% accuracy in 0.19 s	1	Raspberry Pi 4B	N
2023	UniFi [41]	Gesture recognition	99% and 90–98% accuracy for in-domain and cross-domain recognition	1	Widar dataset	N
2023	WiTransformer [42]	Gesture recognition	86.16% accuracy	1	Widar dataset	N
2024	AirFi [43]	Gesture recognition	90% accuracy	1	TP-Link N750 router	N
2024	WiCGesture [44]	Continuous gesture recognition	89.6% for digits and 88.3% for Greek letters	1	Intel 5300	N

**Gesture recognition.** Wireless gesture recognition has emerged as an important part of modern human computer interaction, enabling wide applications including smart home control and virtual reality. Previous studies tried to learn the intricate pattern between signal variation and human gesture under the one-to-one mapping assumption. However, this assumption does not hold, since the received signal is highly dependent on the relative location and orientation of users, as proven by the Fresnel reflection model [18]. Thus, recent works mainly focused on realizing a position-independent robust gesture recognition system, as illustrated in Table 3. Kang et al. [34], Widar 3.0 [39], UniFi [41], WiTransformer [42], and AirFi [43] leverage various deep learning methods, e.g., adversarial learning, multi-view network, and few-shot learning, to realize a robust and efficient recognition. On the other hand, WiGesture [35], HandGest [36], DPSense-WiGesture [37], Niu et al. [38], and WiCGesture [44] attempted to extract distinct and consistent features from a hand-oriented perspective, realizing reliable and continuous recognition either through more fine-grained signal segmentation or signal quality assessment. In addition, WiFine [40] managed to realize real-time gesture recognition using low-end edge devices, e.g., Raspberry Pi. Overall, these methods bring Wi-Fi gesture recognition one step closer to more practical uses.

**Table 4:** Activity recognition.

Year	Reference	Application	Performance	User Number	Device Type	NLOS
2020	Wang et al. [45]	People counting and recognition	86% average accuracy	4	COTS devices	N
2021	Ma et al. [46]	Activity recognition	97% average accuracy	1	Intel 5300	N
2021	MCBAR [47]	Activity recognition	90% average accuracy	1	Atheros chipset	N
2021	WiMonitor [48]	Location and activity monitoring	Not applicable	1	Intel 5300	Y
2022	DeFall [49]	Fall detection	95% detection rate and 1.5% false alarm rate	1	Intel 5300	Y
2022	Ding et al. [50]	Activity recognition	96.85% average accuracy	1	Intel 5300	N
2022	EfficientFi [51]	Activity recognition	98% accuracy	1	TP-Link N750 router	N
2022	TOSS [52]	Activity recognition	82.69% average accuracy	1	Intel 5300	N
2023	FallDar [53]	Fall detection	5.7% false alarm rate and 3.4% missed alarm rate	1	Intel 5300	Y
2023	SHARP [54]	Activity recognition	95% average accuracy	1	ASUS RT-AC86U router	N
2023	Liu et al. [55]	Moving receiver-based activity recognition	10°, 1 cm and 98% accuracy for direction, displacement, and activity estimation	1	COTS WiFi 6 device	N
2023	WiCross [56]	Target passing detection	95% accuracy	1	Intel 5300	N
2024	i-Sample [57]	Activity recognition	10% accuracy gain	1	Intel 5300	N
2024	MaskFi [58]	Activity recognition	97.61% average accuracy	1	TP-Link N750 router	N
2024	MetaFormer [59]	Activity recognition	Improved accuracy in various cross-domain scenarios	1	SiFi, Widar, Wiar datasets	N
2024	SAT [60]	Activity recognition	Improved accuracy and robustness	1	Intel 5300	N
2024	SecureSense [61]	Activity recognition under adversarial attack	Robust performance under various attacks	1	TP-Link N750 router	N
2024	Luo et al. [62]	Activity recognition	98.78% accuracy	1	UT-HAR dataset	N
2024	WiSMLF [63]	Activity recognition	92% average accuracy	1	Intel 5300	N

**Activity recognition.** Wi-Fi-based human activity recognition (HAR) has become the most studied research topic over the past years, covering many applications including people counting [45], fall detection [49,53], door-passing detection [56], and daily activities. Table 4 shows the summary of recent HAR works. Most works tried to address performance degradation due to location, person, and environment dynamics, also known as domain-dependent problems [46,47,50,52,54,57,58,59,62,63]. In addition, WiMonitor [48] studied continuous long-term human activity monitoring, capturing user information such as location change, activity intensity, and time. Moreover, EfficientFi [51] considered the signal transfer-induced communication problem in a large-scale sensing scenario, providing a cloud-enabled solution with efficient CSI compression, while SAT [60] and SecureSense [61] proposed robust sensing schemes under various adversarial attacks. Liu et al. [55] proposed a dynamic Fresnel zone sensing model using a moving receiver such as a smartphone, filling the gap in existing fixed-location transceivers.

**Table 5:** Localization and tracking.

Year	Reference	Application	Performance	User Number	Device Type	NLOS
2022	Niu et al. [64]	Velocity estimation-based tracing	9.38 cm/s, 13.42° and 31.08 cm median error in speed, heading and location estimation	1	Intel 5300	Y
2023	WiTraj [65]	Human walking tracking	2.5% median tracking error	1	Intel 5300	N
2024	FewSense [66]	Tracking	34 cm median error	1	Intel 5300	N
2023	Zhang et al. [67]	Multi-person localization	Sub-centimeter accuracy	1–3	COTS WiFi device + IRS	N
2024	Zhang et al. [68]	Passive localization	0.11 m average error	1	VNA	N
2022	Fan et al. [69]	Moving direction estimation	6.9° median error for moving direction estimation; 16.6° mean error for rotation angle estimation	1	Atheros chipset	Y
2022	Wi-Drone [70]	Tracking-based indoor drone flight control	26.1 cm average location accuracy and 3.8° rotation accuracy	1	AR9580 NICs	N

**Localization and tracking.** Due to the limited channel bandwidth and antenna number of COTS Wi-Fi devices, there have not been many studies on Wi-Fi-based localization and tracking, as shown in Table 5. Recent works tried to improve tracking performance through more accurate target velocity estimations using a moving-induced Doppler Frequency Shift (DFS). Niu et al. [64] optimized velocity estimation by devising a dynamic selection scheme, which can choose the optimal set of receivers for tracking. To better track human walking, WiTraj [65] intelligently combined multi-view information provided by different receivers and differentiated walking with in-place activity to avoid tracking error accumulation. FewSense [66] creatively fused phase and information for better DFS estimation, achieving high accuracy even with fewer CSI samples. In addition to these works, Zhang et al. [67,68] achieved sub-centimeter localization accuracy using the intelligent reflecting surface (IRS) technique. By constructing an IRS, researchers can modulate the spatial distribution of the Wi-Fi signal, improving the spatial resolution of Wi-Fi localization. While promising, their current prototype systems are realized using a vector network analyzer (VNA), requiring further study with a COTS device. Apart from the device-free tracking mentioned above, Fan et al. [69] and Wi-Drone [70] studied device-based tracking applications. Fan et al. [69] obtained accurate moving direction and in-place rotation angle estimation using a single access point, while Wi-Drone [70] realized the first Wi-Fi tracking-based indoor drone flight control system, providing promising possible solutions for indoor localization and navigation.

**Table 6:** Vital sign monitoring.

Year	Reference	Application	Performance	User Number	Device Type	NLOS
2020	MultiSense [71]	Multi-person respiration sensing	0.73 bpm mean error	4	Intel 5300	Y
2021	SMARS [72]	Breath estimation and sleep stage recognition	0.47 bpm median error and 88% accuracy	1	Atheros chipset	Y
2021	WiFi-Sleep [73]	Sleep stage monitoring	81.8% accuracy	1	Intel 5300	N
2021	WiPhone [74]	Respiration monitoring	0.31 bpm average error	1	ASUS RT-AC86U router and Google Nexus 5 smartphone	Y
2022	ResFi [75]	Respiration detection	96.05% accuracy	1	ASUS RT-AC86U router	N
2024	Xie et al. [76]	Respiration sensing with interfering individual	32% mean absolute error reduction	1	VNA or Intel 5300	N

**Vital sign monitoring.** Vital signs play a crucial role in monitoring people’s health and well-being, providing useful information for early prediction and interference with potential diseases. As shown in Table 6, CSI-based vital sign detection mainly focused on respiration estimation. MultiSense [71] studied the multi-person respiration sensing problem, while SMARS [72] and WiFi-Sleep [73] integrated breath monitoring into users’ sleep quality assessment. WiPhone [74] presented a smartphone-based sensing system, achieving robust performance in NLOS scenarios. Xie et al. [76] addressed the motion interference from nearby individuals, bringing respiration monitoring closer to practical application.

**Table 7:** Pose construction and imaging.

Year	Reference	Application	Performance	User Number	Device Type	NLOS
2020	WiPose [77]	Pose construction	2.83 cm average error	1	Intel 5300	N
2020	WiSIA [78]	Target imaging	Not applicable	1	Intel 5300	N
2022	GoPose [79]	3D human pose estimation	4.7 cm accuracy	1 or 2	Intel 5300	Y
2022	Wiffract [80]	Still object imaging	86.7% letter reading accuracy	1	Intel 5300	Y
2023	MetaFi++ [81]	Pose estimation	97.3% for PCK@50	1	TP-Link N750 router	N
2023	WiMeasure [82]	Object size measurement	2.6 mm median error	1	Intel 5300	N
2024	PowerSkel [83]	Pose estimation	96.27% for PCK@50	1	ESP 32 IoT SoC	N
2024	WiProfile [84]	2D target Profiling	1 cm median absolute error	1 target with proper size range	Intel 5300	N

**Pose construction and imaging.** Wi-Fi-based pose estimation and target imaging provides a complementary solution to traditional camera-based perception. As listed in Table 7, WiPose [77], GoPose [79], MetaFi++ [81], and PowerSkel [83] proposed different 3D human skeleton construction frameworks, while WiSIA [78], Wiffract [80], and WiProfile [84] further investigated how to recover target images with Wi-Fi signals. Alternatively, WiMeasure [82] realized millimeter-level high-precision target size measurements, making up for a missing piece of Wi-Fi sensing. It should be noted that in order to achieve fine-grained imaging, the deployment of a high sampling rate and even a customized antenna are usually required, as shown in the subsequent tables. Therefore, Wi-Fi imaging is only applicable for specific application scenarios for the time being.

### 3.2. Wi-Fi Sensing Methodologies

In this section, we divide the related works into three categories, i.e., model-based sensing, hand-crafted statistical pattern extraction-based sensing, and automatic deep pattern extraction-based sensing, as shown from Table 8, Table 9 and Table 10. In each table, “Methodology” briefly describes the specific method adopted, and “Base signal” refers to the sensing signal constructed with raw CSI, including autocorrelation function (ACF), power spectrum density (PSD), Doppler frequency shift (DFS), body-coordinate velocity profile (BVP), and so on. In addition, “Setting” specifies the signal sampling rate required, the number of Tx-Rx pair used, and certain device settings used for the system implementation and performance evaluation.

**Model-based sensing.** Since model-based sensing methods have the clear advantage of interpretability, researchers have developed several models for describing the physical relationship between CSI variation and target behavior, detailed in Section 2. As shown in Table 8, the scattering model has been widely used for velocity and periodic pattern extraction [28,49,72], while the diffraction model has been adopted in near-the-LOS scenarios, i.e., within FFZ, for fine-grained sensing tasks [56,80,82,84]. Although less prevalent in Table 8 [55], the Fresnel zone-based reflection model is in fact the most used model. The reflection model is commonly implicitly incorporated in various sensing systems for quantitatively analyzing signal variations and identifying sensing limitations, thus guiding the implementation of more stable and reliable sensing systems [85,86,87].

**Table 8:** Model-based sensing.

Year	Reference	Methodology	Performance	Base Signal	Sensing Range	Setting
2021	GaitWay [28]	Scattering model	0.12 m median error	ACF of CSI	20 m × 23 m	1500 Hz; single pair of Tx-Rx
2021	SMARS [72]	Scattering model	0.47 bpm median error and 88% accuracy	ACF of CSI	10 m	30 Hz; single pair of Tx-Rx
2022	DeFall [49]	Scattering model	95% detection rate and 1.5% false alarm rate	ACF of CSI	Multi-room	1500 Hz; single pair of Tx-Rx
2022	Wiffract [80]	Keller’s Geometrical Theory of Diffraction	86.7% letter reading accuracy	Power of CSI	1.5 m	Two pairs of Tx-Rx; two-dimensional RX grid synthesis
2023	Liu et al. [55]	Dynamic Fresnel zone model	10°, 1 cm and 98% accuracy for direction, displacement and activity estimation	CSI	Single room	100 Hz; single pair of Tx-Rx
2023	WiCross [56]	Diffraction model-based phase pattern extraction	95% accuracy	CSI ratio	1 m	1000 Hz; single pair of Tx-Rx
2023	WiMeasure [82]	Diffraction model	2.6 mm median error	CSI ratio	Near the LOS path	500 Hz; three pairs of Tx-Rx
2024	WiProfile [84]	Diffraction effect-based profiling + inverse Fresnel transform	1 cm median absolute error	CSI	1.5 m × 1 m	500 Hz; single pair of Tx-Rx; One reference receiving antenna connected to Rx via feeder line

**Hand-crafted statistical pattern extraction-based sensing.** Derived from feature engineering in traditional machine learning processes, researchers have come up with various task-oriented feature extraction schemes, utilizing the in-depth analysis of activity characteristics and advanced signal processing techniques. As shown in Table 9, along with signal processing such as signal segmentation and signal energy estimation, statistical features, such as Doppler frequency shift and speed estimation, motion navigation primitive (MNP), dynamic phase vector (DPV) and motion rotation variable (MRV), have been derived for various sensing tasks. Albeit promising, since feature extraction and selection plays a key role in system performance, hand-crafted features are usually task-specific and not reusable for new tasks, hindering their usage for ubiquitous sensing.

**Table 9:** Hand-crafted statistical pattern-based sensing.

Year	Reference	Methodology	Performance	Base Signal	Sensing Range	Setting
2020	MultiSense [71]	ICA-based BSS	0.73 bpm mean error	Constructed reference-CSI-based signal ratio	4 m × 7.5 m	200 Hz; single pair of Tx-Rx
2020	Wang et al. [45]	Statistical pattern analysis	86% accuracy	PSD of CSI	3.5 m	10 Hz; single pair of Tx-Rx
2021	WiGesture [35]	MNP feature extraction	92.8–94.5% accuracy	CSI ratio	4 m × 7 m	400 Hz; two pairs of Tx-Rx
2021	WiMonitor [48]	Doppler frequency and activity intensity pattern extraction	Not applicable	CSI ratio	Multi-room	200 Hz; single pair of Tx-Rx
2021	WiPhone [74]	Ambient reflection-based pattern extraction	0.31 bpm average error	CSI amplitude	Multi-room apartment	50 Hz; single pair of Tx-Rx with LOS blocked
2022	HandGest [36]	Hand-centric feature extraction, i.e., DPV and MRV	4.7 cm accuracy	CSI ratio	1 m	500 Hz; two pairs of Tx-Rx
2022	Niu et al. [64]	DFS-based velocity estimation + receiver selection	96.05% accuracy	CSI ratio	7 m × 9.8 m	1000 Hz; six pairs of Tx-Rx
2022	Fan et al. [69]	2D-antenna array-based signal parameter estimation	6.9° median error for moving direction estimation; 16.6° mean error for rotation angle estimation	Time-reversal resonating strength of CSI	28 m × 36.5 m	200 Hz; single pair of Tx-Rx; half octagonal array of 6 antennas
2022	Wi-Drone [70]	Rigid-body coordinate transformation-based absolute pose and relative motion estimation	26.1 cm average location accuracy and 3.8° rotation accuracy	CSI	32 m × 18 m	Four pairs of Tx-Rx
2022	DPSense-WiGesture [37]	Signal segmentation + sensing quality-based signal processing	94% average accuracy	CSI	1.2 m	400 Hz; two pairs of Tx-Rx
2022	Niu et al. [38]	Position-independent feature extraction, i.e., movement fragment and relative motion direction change	96% accuracy	CSI ratio	2 m × 2 m	1000 Hz; 2 pairs of Tx-Rx
2022	WiCPD [23]	Feature-based motion, stationary and transition target detector	96.56–100% real-time detection rate	ACF of CSI	Car	30 Hz; single pair of Tx-Rx
2023	Hu et al. [24]	Sub-carrier correlation and covariance feature extraction	95% and 99% true positive rate for distance-based and room-based detection	Power of CSI	Multi-room	30 Hz; single pair of Tx-Rx
2023	WiTraj [65]	DFS extraction + multi-view trajectory estimation + motion detection	2.5% median tracking error	CSI ratio	7 m × 6 m	400 Hz; three pairs of Tx-Rx
2023	Zhang et al. [67]	Intelligent reflecting surface construction	Sub-centimeter accuracy	Received signal power	6 m × 6 m	Single pair of Tx-Rx
2024	Zhang et al. [68]	Intelligent reflecting surface construction	0.11 m average error	Received signal power	3 m × 3 m	Single pair of Tx-Rx
2024	Xie et al. [76]	Respiratory energy-based interference detection and convex optimization-based beam control	32% mean absolute error reduction	CSI	9 m × 6 m	Single pair of Tx-Rx
2024	WiCGesture [44]	Meta motion-based signal segmentation and back-tracking searching-based identification	89.6% for digits and 88.3% for Greek letters	CSI ratio	1 m	400 Hz; Two pairs of Tx-Rx
2024	FewSense [66]	TD-CSI-based Doppler speed estimation	34 cm median error	Time domain CSI difference	7 m × 7 m	1000 Hz; Two pairs of Tx-Rx
2024	WI-MOID [26]	Physical and statistical pattern extraction + SVM + state machine	97.34% accuracy and 1.75% false alarm rate	ACF of CSI	Multi-room	1500 Hz; single pair of Tx-Rx

**Automatic deep pattern extraction-based sensing.** Since it is challenging to devise effective sensing features, more and more studies have begun leveraging various deep learning models for better accuracy and robustness, such as the Convolution Neural Network (CNN) and Recurrent Neural Network (RNN). As seen in Table 10, the combination of CNN and RNN has been widely adopted in recent works [27,30,32,39,73,77,79] due to its advantage in extracting spatial-temporal features from the CSI signal automatically. In addition, to gain more general representation learning, adversarial learning and few-shot learning have also been used for efficient and robust feature training [29,31,34,43,53,57,60,61]. The end-to-end nature of deep learning has made network framework selection and design the primary factor in sensing system implementations.

**Table 10:** Automatic deep pattern-based sensing.

Year	Reference	Methodology	Performance	Base Signal	Sensing Range	Setting
2020	WiPose [77]	CNN + LSTM	2.83 cm average error	3D velocity profile of CSI	Single room	1000 Hz; three pairs of Tx-Rx; distributed deployed receiving Antennas
2020	WiSIA [78]	cGAN	Not applicable	Power of CSI	2.1 m	1000 Hz; two pairs od Tx-Rx; receiving antennas orthogonal to each other
2021	Kang et al. [34]	Adversarial learning and attention scheme	3–12.7% improvement	DFS of CSI	2 m × 2 m	Two pairs of Tx-Rx from Widar dataset
2022	GaitSense [27]	CNN + LSTM + transfer learning + data augmentation	98% accuracy	Gait-BVP of CSI	4.6 m × 4.4 m	1000 Hz; six pairs of Tx-Rx
2021	Ma et al. [46]	CNN + reinforcement learning	97% average accuracy	CSI amplitude	6.8 m × 4 m	100 Hz; single pair of Tx-Rx
2021	MCBAR [47]	GAN and semi-supervised learning	90% average accuracy	CSI amplitude	6.5 m × 6.3 m	single pair of Tx-Rx
2021	WiFi-Sleep [73]	Respiration and movement pattern extraction + CNN-BiLSTM	81.8% accuracy	CSI ratio	Close to the bed	200 Hz; single pair of Tx-Rx
2022	CAUTION [29]	Few-shot learning	93.06 average accuracy	CSI amplitude	5.2 m × 7.2 m	Single pair of Tx-Rx
2022	Ding et al. [50]	DCN + transfer learning	96.85% average accuracy	CSI	6 m × 8 m	200 Hz; single pair of Tx-Rx
2022	EfficientFi [51]	DNN	98% accuracy	CSI amplitude	6.5 m × 5 m	500 Hz; single pair of Tx-Rx
2022	GoPose [79]	2D AOA spectrum + CNN + LSTM	93.2% for 5 users and 76.2% for 11 users	CSI phase	4 m × 4 m	1000 Hz; four pairs of Tx-Rx; L-shaped receiving antennas
2022	ResFi [75]	CNN-based classification	95% accuracy	CSI amplitude	1 m	10 Hz; single pair of Tx-Rx
2022	TOSS [52]	Meta learning + pseudo label strategy	82.69% average accuracy	CSI	Single room	Single pair of Tx-Rx
2022	Widar 3.0 [39]	BVP feature + CNN-RNN	92.7% in-domain and 82.6–92.4% cross-domain accuracy	BVP of CSI	2 m × 2 m	1000 Hz; six pairs of Tx-Rx
2022	WiFine [40]	data enhancement-based feature extraction + lightweight neural network	96.03% accuracy in 0.19 s	CSI	Single room	Single pair of Tx-Rx
2022	Wi-PIGR [30]	Spectrogram optimization + CNN + LSTM	93.5% for single user and 77.15% for 50 users	CSI amplitude	5 m × 5 m	1000 Hz; two pairs of Tx-Rx
2023	Auto-Fi [31]	Geometric self-supervised learning + few-shot calibration	86.83% for gesture; 79.61% for gait	CSI amplitude	Single room	100 Hz; single pair of Tx-Rx
2023	GaitFi [32]	RCN + LSTM + feature fusion	94.2% accuracy	CSI + video	2.1 m	800 Hz; single pair of Tx-Rx
2023	MetaFi++ [81]	CNN + Transformer	97.3% for PCK@50	CSI + video	Single room	1000 Hz; single pair of Tx-Rx
2023	FallDar [53]	Scattering model + VAE generative model + DNN adversarial learning model	5.7% false alarm rate and 3.4% missed alarm rate	ACF of CSI	3.6 m × 8.4 m	1000 Hz; single pair of Tx-Rx
2023	SHARP [54]	Phase correction-based DFS extraction + Nerual network	95% average accuracy	CSI	5 m × 6 m	173 Hz; single pair of Tx-Rx
2023	UniFi [41]	DFS extraction + consistency-guided multi-view deep network + mutual information-based regularization	99% and 90–98% accuracy for in-domain and cross-domain recognition	CSI ratio	2 m × 2 m	Widar dataset
2023	WiTransformer [42]	Transformer	86.16% accuracy	BVP of CSI	2 m × 2 m	Widar dataset
2024	AirFi [43]	Data augmentation + adversarial learning +domain generalization	90% accuracy	CSI amplitude	4 m × 4 m	Single pair of Tx-Rx
2024	i-Sample [57]	Intermediate sample generation + domain adversarial adaptation	10% accuracy gain	CSI	Single room	Single pair of Tx-Rx
2024	MaskFi [58]	Transformer-based encoder + Gate Recurrent Unit network	97.61% average accuracy	CSI + video	Single room	1000 Hz; Single pair of Tx-Rx
2024	MetaFormer [59]	Transformer-based spatial-temporal feature extraction + match-based meta-learning approach	Improved accuracy in various cross-domain scenarios	CSI	Single room	SiFi, Widar, Wiar datasets
2024	PowerSkel [83]	Knowledge distillation network based on collaborative learning and self-attention	96.27% for PCK@50	CSI + Kinect video	Single room	Three pairs of Tx-Rx
2024	SAT [60]	Calibrated confidence-based adversarial sample selection + adversarial learning	Improved accuracy and robustness	CSI	Single room	Single pair of Tx-Rx
2024	SecureSense [61]	Consistency-guided adversarial learning	Robust performance under various attacks	CSI amplitude	5 m × 6.5 m	1000 Hz; single pair of Tx-Rx
2024	Luo et al. [62]	Transformer	98.78% accuracy	CSI	Single room	UT-HAR dataset
2024	Wi-Diag [33]	Independent component analysis-based blind source separation + CycleGAN	87.77% average accuracy	CSI	7 m × 8 m	1000 Hz; single pair of Tx-Rx
2024	WiSMLF [63]	High frequency energy-based sensing scheme selection + VGG/LSTM-based multi-level feature fusion	92% average accuracy	CSI	Single room	100 Hz; single pair of Tx-Rx
2024	Zhu et al. [25]	ResNet18	95.57% average accuracy	Amplified ACF of CSI	6 m × 6.5 m	1500 Hz; single pair of Tx-Rx

Apart from the above differences, we can obtain several additional findings from Table 1, Table 2, Table 3, Table 4, Table 5, Table 6, Table 7, Table 8, Table 9 and Table 10. First, apart from the CSI amplitude and phase information, several new base signals, such as the BVP of CSI, ACF of CSI, and CSI ratio, have been used for alleviating the intrinsic errors of COTS WiFi devices [88]. Among these base signals, the CSI ratio is drawing more attention since it can not only remove the CSI offset, but it can also increase the sensing signal-to-noise rate (SNR) [89]. Second, some works have tried to combine a pattern-based scheme with model-based scheme to ensure the performance and reliability of complex sensing applications. Third, many systems have been developed for single human sensing under constrained deployment, i.e., single room sensing area with the LOS condition satisfied.

## 4. Challenges

Despite the above endeavors devoted to bringing Wi-Fi sensing from laboratory study to real-life applications, either by improving sensing granularity or exploring application scenarios, most of the existing works still face great practical challenges. Specifically, making Wi-Fi sensing system readily available for wide real-world deployment, easily adaptable to different environments, and with enough sensing coverage is of vital importance. This section presents two key challenges faced in existing works, i.e., the domain dependent issue and the sensing range limitation, and it discusses related potential solution explorations.

**Domain dependent issue.** As the superposition result of multi-path signals, Wi-Fi is highly sensitive to various factors, such as locations, orientations, targets, and environments. This is also known as the domain-dependence problem [15,18,86]. For example, the same human activity will lead to quite different CSI variations if the location or orientation of the target changes, as revealed by the Fresnel zone model. Moreover, different sensing environments and device settings will make this inconsistent phenomenon even worse. A sensing system lacking resilience to domain variations is in fact of little practical use for ubiquitous sensing. Thus, in order to make Wi-Fi sensing reusable and robust among different settings, researchers have explored various methods, as summarized in Table 11. Since training effort accounts for a great part of the system deployment cost, Table 11 classifies the related works into three categories, i.e., training-free, training-once, and training + Calibration/Retrained. As seen in Table 11, the training-free scheme is mainly used for simple presence detection tasks [23,24], where a motion-induced threshold is predetermined without training. In addition, for the training-once scheme, the domain-independent feature extraction is the most studied, and it is mainly used along model-based sensing, as listed in Table 8 due to clear interpretability. Moreover, with the increasing complexity of sensing tasks and environments, system recalibration would become inevitable, promoting researchers to reduce the system retraining cost, e.g., utilizing data augmentation, transfer learning, and few-shot learning, as shown in Table 11. It can also be observed that domain-independent feature extraction can be used alone or further integrated with other retraining algorithms. Drawn from the above discussions, for these complex applications, it is expected that combining the strengths of model-based and auto deep learning model-based methods can enable a more general and robust Wi-Fi sensing realization.

**Table 11:** Cross-domain Wi-Fi sensing.

Training Cost	Cross-Domain Scheme	Related Work
Training-free	Domain-independent feature extraction	[23,24]
Training-once	Domain-independent feature extraction	[25,26,27,28,30,34,35,36,37,38,39,41,42,44,49,53,54,64,65,66,72]
Training + Calibration/Retrained	Generative adversarial network	[33,47,53,61]
	Transfer learning	[27,31,34,43,50,57,60]
	Few-shot learning	[29,31,43,52]
	Data augmentation	[27,43,57]
	CNN +LSTM/GRU/Transformer	[25,30,32,39,41,42,46,58,59,62,81]

**Sensing range limitation.** As illustrated in the tables of last section, the existing sensing range is usually just 6–8 m within a single room, while the communication range of Wi-Fi can reach tens of meters, greatly hindering real-world applications. The short sensing range is mainly because Wi-Fi sensing relies on target-induced reflection signal variation, which is much weaker compared to direct LOS signal and contains intrinsic hardware noise. To be more specific, due to hardware imperfections and clock synchronization errors, the raw CSI amplitude contains high impulse and burst noise, while the raw randomly corrupted CSI phase is even more unusable in practice. To deal with this limitation, some researchers proposed employing a new base signal derived from the raw CSI, namely the CSI ratio as seen in Table 8 and Table 9. Defined as the quotient of CSI readings between two receiver antennas, the CSI ratio can remove the amplitude noise and phase noise effectively. More specifically, since different antennas on the same receiver share the same RF chain and clock, the division operation can cancel out most of the noise, gaining a more ideal amplitude and a phase signal with a high signal-to-noise ratio (SNR). The higher SNR and phase usability of the CSI ratio serve as the key enablers for the longer sensing range and higher sensing accuracy. FarSense [90] first increased fine-grained sensing range to 8 m using the CSI ratio signal, while Zeng et al. [91] and DiverSense [92] further boosted the sensing range to 18 m and 40 m by fully utilizing the spatial and frequency diversity. In addition to constructing a new base signal, Wang et al. [93] studied the effect of device placement on sensing SNR and doubly expanded the sensing range by properly placing the transmitter and receiver. Overall, sensing range enlargement is pivoted to large-scale sensing applications and is still in its infancy, requiring further exploration and validation in complex real-world scenario deployments.

## 5. Future Research Trend Discussion

Despite the great effort spent on Wi-Fi sensing over the past years, there still exists a great gap for its pervasive real-life application. Based on the detailed analysis above, we point out three critical barriers that require further research in this section.

**Table 12:** CSI extraction tools.

Year	CSI Extraction Tool	IEEE Standard	Related Work
2011	802.11n CSI Tool [17]	802.11n	[27,28,30,33,35,36,37,38,39,44,46,48,49,50,52,53,56,57,60,63,64,65,66,71,73,77,78,79,80,82,84]
2015	Atheros CSI Tool [94]	802.11n	[29,31,32,47,51,58,61,72,81,94]
2019	Nexmon CSI [95]	802.11 ac	[40,54,74,75,95]
2020	ESP32 CSI Tool [96,97]	Any computer, smartphone or even standalone	[83,96,97]
2021	AX-CSI [98]	802.11 ax	[98]
2022	PicoScenes [99]	802.11 a/g/n/ac/ax	[70,99]

**Table 13:** Wi-Fi sensing datasets.

Year	Dataset	Description	Tool	Related Work
2017	UT-HAR [100]	Activity data	802.11n CSI Tool	[31,46,62]
2018	SignFi [101]	Sign data	802.11n CSI Tool	[40,59]
2018	FallDeFi [102]	Fall data	802.11n CSI Tool	[46,53]
2019	WiAR [103]	Activity and gesture data	802.11n CSI Tool	[59]
2019	Widar [104]	Gesture data	802.11n CSI Tool	[31,34,39,41,42,43,59]
2021	OneFi [105]	Gesture data	802.11n CSI Tool	[105]
2023	MM-Fi [106]	Multi-modal dataset	Atheros CSI Tool	[58]
2023	NTU-Fi [107]	Activity and gait data	Atheros CSI Tool	[62]
2023	SHARP [54]	Activity data	Nexmon CSI	[54]
2023	Cominelli [108]	Activity data	AX-CSI	[108]
2023	WiTraj [65]	Trajectory data	802.11n CSI Tool	[65]

**Sensing assessment standardization.** One key issue is the lack of a standard performance evaluation of the various Wi-Fi sensing systems. Unlike the widely accepted standard evaluation criterion in the computer vision domain, there is still a lack of an effective and consistent testing platform in Wi-Fi sensing. Specifically, the deficiency exists in two aspects, i.e., CSI extraction tool diversity and evaluation dataset scarcity. The diversity of CSI extraction tools is shown in Table 12, with Intel 5300 NIC-based 802.11n CSI Tool being the most popular one used. However, sensing techniques developed with old 802.11n protocol have not explored the innovations of newer standards and may even fail when used on new-generation Wi-Fi cards [108,109]. In addition, as illustrated in Table 13, although there have been some publicly released datasets, none of them have been widely used. Existing works mostly adopt self-collected datasets, collected in different scenarios with different tools, hindering the comparability and replicability of research outcomes. To build comprehensive datasets without labor-intensive and time-consuming efforts, researchers have studied radio signal synthesis [110,111] and physical data augmentation [112], providing promising solutions to the data scarcity problem. We believe a more unified CSI extraction tool compatible with the new 802.11 standard and a set of standard datasets for a benchmark comparison should be indispensable for the further research cooperation and development of Wi-Fi sensing.

**Sensing and communication balance.** As illustrated in Table 14, most sensing systems require a high sampling rate for reliable performance, which interferes with regular Wi-Fi communication. To be more specific, the data throughput undergoes great drop when the sampling rate for sensing is higher than 50 Hz [66]. SenCom [113] managed to extract CSI from general communication packets and obtained evenly sampled and sufficient CSI data with a detailed signal processing technique. While appealing, SenCom is not yet applicable for COTS clients. Thus, the ways of enabling Wi-Fi sensing while maintaining communication capability, i.e., achieving sensing and communication balance, remain an open problem in the current ISAC area.

**Sensing generalization and reliability.** As noted in Table 12, raw CSI reading is still only accessible with limited hardware; some researchers resorted to sensing with other Wi-Fi signals. For instance, since the beamforming feedback matrix (BFM) is readily available with all new-generation MU-MIMO-enabled Wi-Fi cards, researchers have explored generalized Wi-Fi sensing using BFM [114,115]. In addition, to improve the reliability of sensing, multi-modal sensing, which integrates Wi-Fi and other sensing modalities, e.g., video [32,52,81,116] and received signal strength indicator (RSSI) [117], are worth further studying.

**Table 14:** Sampling rate of recent works.

Sampling Rate	Related Work
≤100 Hz	[23,24,31,45,46,55,63,66,72,74,75,83]
100 Hz–500 Hz	[35,36,37,44,48,50,51,54,65,71,73,82,84]
>500 Hz	[25,26,27,28,30,32,33,38,39,49,53,56,58,64,77,78,79,81]

Apart from the above discussion, the physical challenges of the existing Wi-Fi infrastructure should also be noticed, which will greatly determine the possible sensing limit of Wi-Fi sensing. First, due to hardware and network design, clock asynchronism between Wi-Fi transmitter and receiver is a severe issue in an ISAC system. It introduces a time-varying random phase offset in raw CSI, making reliable feature extraction difficult. Second, except for target influence, dynamic parameter adjustments of the network card during transmission also affect the CSI measurement, which is highly dependent on the hardware design. Third, large-scale Wi-Fi sensing needs to obtain CSI from multiple distributed receivers. The ways of enabling CSI estimation and alignment over multiple devices are a challenging problem. Currently, there is no universal solution to the above challenges, requiring cooperative efforts from application researchers, chip manufactures, and communication equipment vendors.

## 6. Conclusions

Owing to the active participation from numerous researchers, notable advances have been made in Wi-Fi sensing techniques in recent years. In an effort to gain insight into future trends, this paper reviews major achievements over the last 5 years and carries out an in-depth analysis of various methods, including limitations and practical challenges faced in existing systems. Moreover, to realize massive real-life applications, this paper highlights three imperative and promising future directions which are as follows: sensing assessment standardization, sensing and communication balance, and sensing generalization and reliability. We hope this review can help people better understand the progress and problems within the current Wi-Fi sensing research field, inspiring more amazing ideas for the upcoming ubiquitous ISAC.

## Data Availability

Not applicable.

## Appendix A

Table A1 summaries all references mentioned in Section 3, pointing out corresponding application and methodology categories, and can direct the interested reader to related subsections for a more detailed description.

**Table A1:** Summary of references in Section 3.

Year	Reference	Application	Methodology	Related Subsections
2022	WiCPD [23]	In-car child presence detection	Feature-based motion, stationary and transition target detector	Table 1 in Section 3.1; Table 9 in Section 3.2
2023	Hu et al. [24]	Proximity detection	Sub-carrier correlation and covariance feature extraction	Table 1 in Section 3.1; Table 9 in Section 3.2
2024	Zhu et al. [25]	Human and non-human differentiation	ResNet18	Table 1 in Section 3.1; Table 10 in Section 3.2
2024	WI-MOID [26]	Edge device-based human and non-human differentiation	Physical and statistical pattern extraction + SVM + state machine	Table 1 in Section 3.1; Table 9 in Section 3.2
2021	GaitSense [27]	Gait-based human identification	CNN + LSTM + transfer learning + data augmentation	Table 2 in Section 3.1; Table 10 in Section 3.2
2021	GaitWay [28]	Gait speed estimation	Scattering model	Table 2 in Section 3.1; Table 8 in Section 3.2
2022	CAUTION [29]	Gait-based human authentication	Few-shot learning	Table 2 in Section 3.1; Table 10 in Section 3.2
2022	Wi-PIGR [30]	Gait recognition	Spectrogram optimization + CNN + LSTM	Table 2 in Section 3.1; Table 10 in Section 3.2
2023	Auto-Fi [31]	Gesture and gait recognition	Geometric self-supervised learning + few-shot calibration	Table 2 in Section 3.1; Table 10 in Section 3.2
2023	GaitFi [32]	Gait recognition	RCN + LSTM + feature fusion	Table 2 in Section 3.1; Table 10 in Section 3.2
2024	Wi-Diag [33]	Multi-subject abnormal gait diagnosis	Independent component analysis-based blind source separation + CycleGAN	Table 2 in Section 3.1; Table 10 in Section 3.2
2021	Kang et al. [34]	Gesture recognition	Adversarial learning and attention scheme	Table 3 in Section 3.1; Table 10 in Section 3.2
2021	WiGesture [35]	Gesture recognition	MNP feature extraction	Table 3 in Section 3.1; Table 9 in Section 3.2
2022	HandGest [36]	Handwriting recognition	Hand-centric feature extraction, i.e., DPV and MRV	Table 3 in Section 3.1; Table 9 in Section 3.2
2022	DPSense-WiGesture [37]	Gesture recognition	Signal segmentation + sensing quality-based signal processing	Table 3 in Section 3.1; Table 9 in Section 3.2
2022	Niu et al. [38]	Gesture recognition	Position-independent feature extraction, i.e., movement fragment and relative motion direction change	Table 3 in Section 3.1; Table 9 in Section 3.2
2022	Widar 3.0 [39]	Cross-domain gesture recognition	BVP feature + CNN-RNN	Table 3 in Section 3.1; Table 10 in Section 3.2
2022	WiFine [40]	Gesture recognition	Data enhancement-based feature extraction + lightweight neural network	Table 3 in Section 3.1; Table 10 in Section 3.2
2023	UniFi [41]	Gesture recognition	DFS extraction + consistency-guided multi-view deep network + mutual information-based regularization	Table 3 in Section 3.1; Table 10 in Section 3.2
2023	WiTransformer [42]	Gesture recognition	Transformer	Table 3 in Section 3.1; Table 10 in Section 3.2
2024	AirFi [43]	Gesture recognition	Data augmentation + adversarial learning + domain generalization	Table 3 in Section 3.1; Table 10 in Section 3.2
2024	WiCGesture [44]	Continuous gesture recognition	Meta motion-based signal segmentation and back-tracking searching-based identification	Table 3 in Section 3.1; Table 9 in Section 3.2
2020	Wang et al. [45]	People counting and recognition	Statistical pattern analysis	Table 4 in Section 3.1; Table 9 in Section 3.2
2021	Ma et al. [46]	Activity recognition	CNN + reinforcement learning	Table 4 in Section 3.1; Table 10 in Section 3.2
2021	MCBAR [47]	Activity recognition	GAN and semi-supervised learning	Table 4 in Section 3.1; Table 10 in Section 3.2
2021	WiMonitor [48]	Location and activity monitoring	Doppler frequency and activity intensity pattern extraction	Table 4 in Section 3.1; Table 9 in Section 3.2
2022	DeFall [49]	Fall detection	Scattering model	Table 4 in Section 3.1; Table 8 in Section 3.2
2022	Ding et al. [50]	Activity recognition	DCN + transfer learning	Table 4 in Section 3.1; Table 10 in Section 3.2
2022	EfficientFi [51]	Activity recognition	DNN	Table 4 in Section 3.1; Table 10 in Section 3.2
2022	TOSS [52]	Activity recognition	Meta learning + pseudo label strategy	Table 4 in Section 3.1; Table 10 in Section 3.2
2023	FallDar [53]	Fall detection	Scattering model + VAE generative model + DNN adversarial learning model	Table 4 in Section 3.1; Table 10 in Section 3.2
2023	SHARP [54]	Activity recognition	Phase correction-based DFS extraction + Nerual network	Table 4 in Section 3.1; Table 10 in Section 3.2
2023	Liu et al. [55]	Moving receiver-based activity recognition	Dynamic Fresnel zone model	Table 4 in Section 3.1; Table 8 in Section 3.2
2023	WiCross [56]	Target passing detection	Diffraction model-based phase pattern extraction	Table 4 in Section 3.1; Table 8 in Section 3.2
2024	i-Sample [57]	Activity recognition	Intermediate sample generation + domain adversarial adaptation	Table 4 in Section 3.1; Table 10 in Section 3.2
2024	MaskFi [58]	Activity recognition	Transformer-based encoder + Gate Recurrent Unit network	Table 4 in Section 3.1; Table 10 in Section 3.2
2024	MetaFormer [59]	Activity recognition	Transformer-based spatial-temporal feature extraction + match-based meta-learning approach	Table 4 in Section 3.1; Table 10 in Section 3.2
2024	SAT [60]	Activity recognition	Calibrated confidence-based adversarial sample selection + adversarial learning	Table 4 in Section 3.1; Table 10 in Section 3.2
2024	SecureSense [61]	Activity recognition under adversarial attack	Consistency-guided adversarial learning	Table 4 in Section 3.1; Table 10 in Section 3.2
2024	Luo et al. [62]	Activity recognition	Transformer	Table 4 in Section 3.1; Table 10 in Section 3.2
2024	WiSMLF [63]	Activity recognition	High-frequency energy-based sensing scheme selection + VGG/LSTM-based multi-level feature fusion	Table 4 in Section 3.1; Table 10 in Section 3.2
2022	Niu et al. [64]	Velocity estimation-based tracing	DFS-based velocity estimation + receiver selection	Table 5 in Section 3.1; Table 9 in Section 3.2
2023	WiTraj [65]	Human walking tracking	DFS extraction + multi-view trajectory estimation + motion detection	Table 5 in Section 3.1; Table 9 in Section 3.2
2024	FewSense [66]	Tracking	TD-CSI-based Doppler speed estimation	Table 5 in Section 3.1; Table 9 in Section 3.2
2023	Zhang et al. [67]	Multi-person localization	2D antenna array-based signal parameter estimation	Table 5 in Section 3.1; Table 9 in Section 3.2
2024	Zhang et al. [68]	Passive localization	Rigid-body coordinate transformation-based absolute pose and relative motion estimation	Table 5 in Section 3.1; Table 9 in Section 3.2
2022	Fan et al. [69]	Moving direction estimation	Intelligent reflecting surface construction	Table 5 in Section 3.1; Table 9 in Section 3.2
2022	Wi-Drone [70]	Tracking-based indoor drone flight control	Intelligent reflecting surface construction	Table 5 in Section 3.1; Table 9 in Section 3.2
2020	MultiSense [71]	Multi-person respiration sensing	ICA-based BSS	Table 6 in Section 3.1; Table 9 in Section 3.2
2021	SMARS [72]	Breath estimation and sleep stage recognition	Scattering model	Table 6 in Section 3.1; Table 8 in Section 3.2;
2021	WiFi-Sleep [73]	Sleep stage monitoring	Respiration and movement pattern extraction + CNN-BiLSTM	Table 6 in Section 3.1; Table 10 in Section 3.2
2021	WiPhone [74]	Respiration monitoring	Ambient reflection-based pattern extraction	Table 6 in Section 3.1; Table 9 in Section 3.2
2022	ResFi [75]	Respiration detection	CNN-based classification	Table 6 in Section 3.1; Table 10 in Section 3.2
2024	Xie et al. [76]	Respiration sensing with interfering individual	Respiratory energy-based interference detection and convex optimization-based beam control	Table 6 in Section 3.1; Table 9 in Section 3.2
2020	WiPose [77]	Pose construction	CNN + LSTM	Table 7 in Section 3.1; Table 10 in Section 3.2
2020	WiSIA [78]	Target imaging	cGAN	Table 7 in Section 3.1; Table 10 in Section 3.2
2022	GoPose [79]	3D human pose estimation	2D AOA spectrum + CNN + LSTM	Table 7 in Section 3.1; Table 10 in Section 3.2
2022	Wiffract [80]	Still object imaging	Keller’s Geometrical Theory of Diffraction	Table 7 in Section 3.1; Table 8 in Section 3.2
2023	MetaFi++ [81]	Pose estimation	CNN + Transformer	Table 7 in Section 3.1; Table 10 in Section 3.2
2023	WiMeasure [82]	Object size measurement	Diffraction model	Table 7 in Section 3.1; Table 8 in Section 3.2
2024	PowerSkel [83]	Pose estimation	Knowledge distillation network based on collaborative learning and self-attention	Table 7 in Section 3.1; Table 10 in Section 3.2
2024	WiProfile [84]	2D target Profiling	Diffraction effect-based profiling + inverse Fresnel transform	Table 7 in Section 3.1; Table 8 in Section 3.2
